# Functions of post-conflict bystander affiliations toward aggressors and victims in bottlenose dolphins

**DOI:** 10.1038/s41598-020-60423-6

**Published:** 2020-03-02

**Authors:** Chisato Yamamoto, Toshiaki Ishibashi, Nobuyuki Kashiwagi, Masao Amano

**Affiliations:** 1 Primate Research Institute, Kyoto University, Inuyama, Aichi 484-8506 Japan; 2 Graduate School of Fisheries and Environmental Sciences, Nagasaki University, 1-14 Bunkyo-machi, Nagasaki, 852-8521 Japan; 3 Shimonoseki Marine Science Museum, 6-1 Arcaport, Shimonoseki, Yamaguchi, 750-0036 Japan; 4 Kagoshima City Aquarium, 3-1 Honkoushinmachi, Kagoshima, 892-0814 Japan

**Keywords:** Behavioural ecology, Animal behaviour

## Abstract

Post-conflict affiliations initiated by bystanders (bystander affiliation) toward aggressors or victims have been suggested to represent the function of conflict management in some social living species. However, the function of bystander affiliations toward aggressors and victims has not been examined in marine mammals. In the present study, we investigated the function of bystander affiliations to aggressors and victims in bottlenose dolphins: self-protection, the substitute of reconciliation, social facilitation and tension relief of opponents. These bystander affiliations did not reduce post-conflict attacks by former opponents against group members. Bystander affiliation to aggressors tended to be performed by a bystander who had an affiliative relationship with the aggressor but not with the victim. Bystander affiliation to victims also tended to be initiated by a bystander who had an affiliative relationship with the victim but not the aggressor and was close to former opponents at the end of aggressions. Affiliation among group members who stayed near former opponents during aggressions did not increase after aggressions compared to that under control conditions. Renewed aggressions between former opponents decreased after bystander affiliations in our previous study. Bystanders who showed social closeness to former opponents may initiate bystander affiliation toward their affiliative former opponents because they may feel emotion, such as anxiety and excitement, of former opponents. Bystander affiliation toward aggressors and victims may function as tension relief between former opponents. Bystanders of bottlenose dolphins, who may have a relaxed dominant style, might initiate post-conflict affiliation to affiliative individuals unaffected by the dominance relationships among them, unlike despotic species.

## Introduction

Animals which live in social groups often experience aggression. Aggression involves various costs, such as damage to social relationships, increasing the stress, increasing risk of receiving attack, the use of time and energy and risk of injury. Strategies that reduce aggression costs, called conflict management, include post-conflict affiliation between former opponents and post-conflict affiliation initiated by bystanders toward former opponents^1^. In several primates and non-primates, post-conflict affiliation is sometimes offered to former opponents by bystanders who do not participate in aggression^1^. A key area of research is focused the function of post-conflict affiliation initiated by bystanders^2,3^, with several possible function being suggested for bystander affiliations in several animals.

The first possible function is self-protection by bystanders. Bystanders are likely to be attacked by aggressors and victims after the original aggressions^4–6^. The probability of receiving post-conflict attacks from former opponents decreased when bystander affiliation occurred^7–11^. In contrast, self-protection from attack by a victim toward group members was not supported in Barbary macaques (*Macaca sylvanus*) because the probability of post-conflict attacks by victims did not increase toward group members^12^. If self-protection function is correct in our bottlenose dolphins, attacks by former opponents toward group members should increase after the original aggression, and bystander affiliations should reduce such attacks.

A second possible function is the substitution of reconciliation. Reconciliation (post-conflict affiliation between former opponents) restores social relationships between former opponents, because this affiliation reduced renewed aggression between former opponents and post-conflict stress^13^. However, because approaching the opponent is sometimes risky as it might lead to renewed attacks, reconciliation was likely to occur when former opponents shared a valuable relationship such as kin or friend, or the probability of renewed aggression was low^13^. When reconciliation does not seem to occur, the bystander may offer post-conflict affiliation toward the enemy on behalf of their kin or friend. These affiliations may repair the relationship between former opponents^14–16^. If this function is correct, bystanders should initiate affiliation toward the enemy of their affiliative partner before reconciliation occurred, and bystander affiliation should have the same function as reconciliation, including reducing renewed aggressions and post-conflict stress.

A third possible function is social facilitation^17^. Bystanders may initiate affiliation toward former opponents to a nearby individual just because they are aroused by conflict, especially in non-despotic species that approach former opponents as bystanders for facilitation. If this function is correct, physical closeness to—rather than an affiliative relationship with—former opponents was likely to influence the occurrence of bystander affiliation if the animals have non-despotic relationships. In addition, if physical closeness with former opponents promotes affiliation immediately after aggression, affiliation among group members who stay near former opponents increases after aggressions.

The tension relief of former opponents is another possible function. Social tension between former opponents increased after the end of aggressions, as well as during aggressions^18^. The aggressive tendency of the aggressor was high after the aggressions, and the aggressor tended to attack the victim after the original attack^13^. In addition, both the aggressors and victims remained anxious after aggressions because of the deterioration of the relationship and the increased risk of receiving attacks^13^. Bystander affiliation toward aggressors may function to reduce the aggressive tendency of aggressors^9,19^, and bystander affiliation toward victims may function to reduce anxiety^19,20^. Bystanders who have more affiliative relationships with victims are likely to perform bystander affiliation toward victims, because individuals may receive the anxiety of a socially close partner^20^. If bystander affiliation has the function of relieving the tension of former opponents, and because the aggressive tendency and risk of receiving attacks increases after the aggressions, bystanders would initiate post-conflict affiliation toward former opponents who shared an affiliative relationship with them to reduce the likelihood that they initiate renewed attacks on their opponent or receive them.

Although the functions of bystander affiliations to aggressors or victims have been investigated in some terrestrial animals, especially primates, it has not yet been investigated in marine mammals. Bottlenose dolphins (*Tursiops truncatus*) live in a fission–fusion society in which group members change frequently^21^. Adult females associate with most other females in the population, but they are likely to have a relatively stable relationship with specific females. Mother and calf have a strong relationship for three to six years after birth. Adult males are likely to form pair or trio that have strong bonds. In despotic dominance species, dominant individuals usually attack lower-ranked individuals unilaterally and aggressors usually initiate friendly reunions toward victims after the previous aggression as the approach of victim toward aggressor is risky^22,23^. In bottlenose dolphins, victims attack aggressors frequently and they approach the aggressors for friendly reunions, suggesting that they show relaxed dominant style^24^.

Bottlenose dolphins communicate using vocalization with tonal calls and pulsed calls. Whistles are tonal calls with a frequency modulated pure tone and a narrow band. One of the suggested functions of whistles is as a distress call. The whistle rate has been reported to increase during a temporary capture compared to that under undisturbed conditions^25,26^. When one dolphin sank in the water and produced a whistle while emitting a bubble stream, other dolphins helped her to swim and breathe^27^. Burst-pulses are one of the pulsed calls with a broadband sound. Dolphins often produced burst-pulses while expelling bubble during aggressions, suggesting that burst-pulses in aggressions function as a threat^28,29^.

Bottlenose dolphins demonstrate post-conflict affiliation between former opponents and post-conflict affiliation initiated by bystanders toward aggressors or victims. As post-conflict affiliation between former opponents reduced renewed aggression between former opponents, post-conflict affiliation between former opponents may function as reconciliation^30^. Bystander affiliation toward aggressors and victims reduced renewed aggressions by both aggressors and victims^30^. However, previous study did not investigate other points, such as the effect of bystander affiliation on post-conflict attacks by former opponents on group members, or how bystanders have relationships with former opponents. We examined which function of bystander affiliation toward aggressors and victims was the most likely for bottlenose dolphins: self-protection, substitute of reconciliation, social facilitation, and tension relief of former opponents.

## Results

### Post-conflict attacks by former opponents to group members and the effect of bystander affiliation

To investigate whether attacks by former opponents to group members increase after the original aggression, the probability of attacks by former opponents to group members was compared between unaffiliated post-conflict (PC) and matched-control (MC) subjects using generalized linear mixed model (GLMM). The PC period was set as 10 min after the original aggression. PC was labelled as unaffiliated PC when there were no affiliative behaviors (flipper-rubbing, contact swimming, and synchronized swimming) with the former opponent during PC. MC period was similar to the next possible observation day for matched-PC (see Method). The occurrence of attacks by aggressors toward other group members did not differ between unaffiliated PCs and MCs (GLMM: *N* = 128 unaffiliated PC–MC, *β* = 1.24, *SE* = 0.87, *odd ratio* = 3.49, full versus null model: *χ*^*2*^ = 2.39, *df* = 1, *P* = 0.12). The probability of attacks by victims toward group members in unaffiliated PCs did not differ from that in MCs (GLMM: *N* = 124 unaffiliated PC–MC, *β* = 1.09, *SE* = 0.70, *odd ratio* = 3.00, full versus null model: *χ*^*2*^ = 2.63, *df* = 1, *P* = 0.10).

To investigate the effect of bystander affiliation on PC attacks on group members, the probability of attack by former opponents on group members was compared between unaffiliated PCs and the periods after bystander affiliation. There were no differences in PC attack frequency by aggressors between unaffiliated PCs and after bystander affiliation to aggressors (GLMM: *N* = 104 unaffiliated PC and period after bystander affiliation, *β* = 1.36, *SE* = 1.13, *odd ratio* = 3.10, full versus null model: *χ*^*2*^ = 1.83, *df* = 1, *P* = 0.18). The probability of PC attacks by victim did not differ between unaffiliated PCs and periods after bystander affiliation toward victim (GLMM: *N* = 102 unaffiliated PC and period after bystander affiliation, *β* = 1.56, *SE* = 1.09, *odd ratio* = 2.76, full versus null model: *χ*^*2*^ = 2.83, *df* = 1, *P* = 0.09).

### Effect of factors on the occurrence of bystander affiliation

We investigated whether the occurrence of bystander affiliation was affected by (1) the occurrence of other post-conflict affiliation types, (2) the duration of aggression, (3) the occurrence of counterattack (whether former opponents attacked each other during the original aggression), and (4) whether the aggressor or victim is a calf. Characteristics of aggressions and former opponents were affected by the occurrence of post-conflict affiliation^18^. There was independency between post-conflict affiliations^31^. When post-conflict affiliation between former opponents (reconciliation) occurred before bystander affiliation, we recorded that reconciliation occurred in this PC. We recorded whether bystander affiliation toward the opponent occurred before bystander affiliation toward the other former opponent. Bystander affiliation toward aggressors was less likely to occur after reconciliation or bystander affiliation toward victims, but this affiliation was not influenced by the aggression duration or direction, and whether one or both former opponents were calves (Table 1a). Similarly, bystander affiliation toward victims was less likely to occur when reconciliation or bystander affiliation toward aggressors occurred, but other factors did not influence this affiliation (Table 1b).

**Table 1:** Generalized linear mixed model results for effect of factors on bystander affiliation occurring.

	β	SE	P	Odds ratio
(a) Bystander affiliation toward aggressors (*N* = 347 PC)				
Occurrence of reconciliation	−1.03	0.35	0.004	0.36
Occurrence of bystander affiliation toward victims	−0.84	0.33	0.01	0.43
Duration of aggressions	−0.003	0.003	0.24	1.00
Counter aggressions	−0.39	0.28	0.15	0.68
Aggressor is calf	0.42	0.57	0.46	1.52
Victim is calf	−0.37	0.39	0.33	0.70
Full versus null model comparison: χ^2^ = 20.98, df = 6, P = 0.002				
(b) Bystander affiliation toward victim (*N* = 345 PC)				
Occurrence of reconciliation	−1.24	0.38	0.001	0.29
Occurrence of bystander affiliation toward aggressors	−0.84	0.33	0.011	0.43
Duration of aggressions	−0.003	0.003	0.27	1.00
Counter aggressions	−0.32	0.29	0.27	0.73
Aggressor is calf	0.05	0.53	0.92	1.05
Victim is calf	0.31	0.64	0.63	1.36
Full versus null model comparison: χ^2^ = 20.16, df = 6, P = 0.003				

### Effect of affiliative relationships and physical closeness on bystander affiliations

We investigated the effect of affiliative relationships between the enemy and bystander on the frequency of bystander affiliation. To elucidate affiliative relationship, we calculated the affiliative index using the frequency of synchronized swimming (one of affiliation types) during periods excluding after aggressions. The frequency of bystander affiliation toward aggressor was not influenced by the affiliative index between bystander and victim (GLMM: *N* = 80 victim–bystander pair, *β* = −0.38, *SE* = 0.69, *odd ratio* = 0.68, full versus null model: *χ*^*2*^ = 0.30, *df* = 1, *P* = 0.58). The affiliative index between bystander and aggressor did not affect the frequency of bystander affiliation toward victims occurring (GLMM: *N* = 78 aggressor–bystander pair, *β* = 0.34, *SE* = 0.59, *odd ratio* = 1.40, full versus null model: *χ*^*2*^ = 0.30, *df* = 1, *P* = 0.58).

We investigated whether the frequency of bystander affiliation was affected by the affiliative relationship between former opponent and bystander, physical closeness with former opponents at the end of the aggression, and whether the bystander was a calf using GLMM. For physical closeness, we recorded individuals who were closest to former opponents at the end of the aggression. Bystander affiliation toward aggressors was likely to be performed by bystanders who had more affiliative relationships with aggressors, but the frequency of bystander affiliation toward aggressors was not affected by the fact that the bystander was a calf nor by the physical closeness with former opponents at the end of aggressions (Table 2a). Bystanders who have more affiliative relationships with victims and stay nearer to former opponents were more likely to initiate post-conflict affiliation toward victims (Table 2b).

**Table 2:** Generalized linear mixed model results for factors affecting bystander affiliation frequency.

	β	SE	P	Odds ratio
(a) Bystander affiliation toward aggressors (*N* = 77 aggressor-bystander pair)				
Affiliative index between aggressor and bystander	3.21	0.62	<0.001	24.78
Physical closeness with former opponents	1.29	0.81	0.11	3.63
Bystander is calf	−0.48	0.41	0.25	0.62
Full versus null model comparison: χ^2^ = 56.15, df = 3, P < 0.001				
(b) Bystander affiliation toward victim (*N* = 82 victim-bystander pair)				
Affiliative index between victims and bystander	2.89	0.52	<0.001	18.00
Physical closeness with former opponents	1.28	0.52	0.01	3.60
Bystander is calf	−0.08	0.41	0.85	0.92
Full versus null model comparison: χ^2^ = 37.34, df = 3, P < 0.001				

### Occurrence of post-conflict affiliation between group members

We investigated whether group members who stayed near the former opponents at the end of aggressions perform affiliation with members other than former opponents using the PC–MC method. Each PC–MC pair was classified into three categories. If affiliation started earlier in PC than in MC or occurred in only PC, the PC–MC pair was labeled as “attracted.” If affiliation started earlier in MC than in PC or occurred in only MC, the PC–MC pair was labeled as “dispersed.” If affiliation started at the same time or did not occur in PC and MC, the PC–MC pair was labeled as “neutral.” The proportion of attracted pairs did not significantly differ from that of dispersed pairs (mean ± SE: attracted, 17.6 ± 4.9%: dispersed, 30.4 ± 7.5%: Wilcoxon matched-pairs singed rank exact test: *N* = 14, *V* = 13, *P* = 0.17).

### Vocalization and bubbles emitted during aggressions

To investigate whether former opponents demonstrated excitement and anxiety during aggression, we recorded the whistles, burst-pulse vocalizations, and bubbles emitted. The emission of bubble streams helped us to identify the sounding dolphin^28,29,32,33^. Whistles were recorded during 63 of 81 aggressions, while burst-pulse vocalizations were recorded in 61 of 81 aggressions. The emission of bubble streams by aggressors was observed in 44 of the burst-pulses and six of the whistles. Bubble stream emission by victims was observed in 17 of the burst-pulses and 20 of the whistles.

## Discussion

The present study examined four possible functions of bystander affiliations toward aggressors or victims in bottlenose dolphins: self-protection, substitute of reconciliation, social facilitation and tension relief. First, if the self-protection function is correct, attack by a former opponent to group members (not the opponent) should increase after the original aggression and it decrease when bystander affiliation occurs^8^. However, in bottlenose dolphins, attacks by aggressors toward group members did not increase after the original aggression. The probability of PC attacks by aggressors did not decrease after bystander affiliation toward aggressors compared to that in unaffiliated PC. Similarly, PC attacks by victims toward group members did not increase in PC periods. The probability of PC attacks by victims after bystander affiliation toward victims did not differ from that in unaffiliated PC. These results did not support the self-protection function by bystanders.

The second possible function is the substitute of reconciliation^14^, which proposes that bystander affiliations occur when reconciliation does not happen, and are offered by bystanders who have affiliative relationships with the enemy of their affiliative partner (for example, bystander affiliation to aggressors is offered by bystanders who have affiliative relationships with the victims), and demonstrate the same function as reconciliation. Bottlenose dolphins performed bystander affiliation toward aggressors more frequently when reconciliation did not occur. Bystander affiliations toward aggressors as well as reconciliation, reduced renewed aggression between former opponents^30^. However, bystanders who had an affiliative relationship with the victim tended not to initiate bystander affiliation toward aggressors. Similarly, although bystander affiliation toward victims occurred more frequently when reconciliation did not happen and showed the same function as reconciliation (i.e., reduced renewed aggression between former opponents), it did not tend to be initiated by bystanders who had the affiliative relationships with aggressors. Thus, our findings did not support the substitute of reconciliation function.

The third possible function is social facilitation^17^. This proposes that bystanders who stay near to former opponents are likely to initiate post-conflict affiliation in a relaxed society. If physical closeness with former opponents was aroused to initiate affiliation, affiliation between group members who stay near former opponents during the aggressions increased after aggressions. The fact that female bottlenose dolphins may have a relaxed society^24^ supported this function. However, physical closeness to former opponents did not affect the frequency of bystander affiliation toward aggressors. Affiliation between group members did not increase after aggressions. These results suggest that bystander affiliation toward aggressors did not support the social facilitation function. In bystander affiliation toward victims, physical closeness and an affiliative relationship with victims increased the opportunity of initiating post-conflict affiliation. However, affiliation between group members did not increase during and after aggressions, even if they stayed close to former opponents during aggressions. These results suggested that an affiliative relationship, rather than physical closeness, promoted the initiation of affiliation after aggressions. Therefore, our results of bystander affiliation toward victims did not fully support the social facilitation function.

The fourth function is the tension relief of former opponents. If this function is correct, the aggressive tendency and anxiety of former opponents is increased by aggressions; bystanders initiate post-conflict affiliation toward former opponents who shared affiliative relationships with them because individuals may receive the anxiety of socially close partners^20^, and bystander affiliation reduces renewed aggression. The aggressors often produced burst-pulses with bubble streams during the aggressions. The aggressors attacked the victim more frequently in the period after the original aggressions than in that of MC^30^. These results suggest that aggressors retain aggressive tendency toward their victims after the end of aggressions. In addition, aggressors rarely produced whistles during the aggressions. The risk that aggressors receive attacks from victims was high after the aggressions, and relationships between former opponents may be damaged by the aggressions^30^. These findings suggest that aggressors feel the anxiety of the aggressions, which is in accordance with a previous report on primates^13^. Bystander affiliation toward aggressors reduced renewed aggressions by aggressors, suggesting that the aggressive tendency of aggressors was alleviated by bystander affiliation^30^. Renewed attacks by victims toward aggressors was also reduced by bystander affiliation toward aggressors^30^. Although our study did not measure the anxiety directly, this result might show the indirect alleviation of aggressors’ anxiety. Bystander affiliation toward aggressors tended to be initiated by bystanders who shared affiliative relationships with them. To burst-pulsed vocalization and whistling by aggressors may serve as a signal to transmit their emotion to the group members, bystanders who were socially close with aggressors may feel the aggressors’ tension growing. Our results support the function of tension relief for bystander affiliation toward aggressors. Victims sometimes produced burst-pulses with bubble streams. Victims often attacked the aggressors during the original aggressions and after the aggressions^30^. These results indicated that the aggressive tendency of the victims was still high after the end of aggressions. The victims produced whistles with bubble streams frequently during aggressions. The victims were likely to receive attacks from the aggressors in PC, and the relationship with aggressors may be damaged by the aggressions^30^. These findings suggest that the victims feel anxiety from the aggressions. However, renewed aggressions initiated by aggressors and victims decreased after bystander affiliation toward victims^30^. Bystanders who shared affiliative relationships with victims tended to initiate bystander affiliation toward them. Bystander affiliation toward victims may also function as tension relief for victims. In conclusion, bystander affiliation toward aggressors and victims may function as tension relief between former opponents. Bystanders who were socially close to former opponents may initiate bystander affiliation toward their affiliative former opponent because they may be sensitive to the anxiety and excitement of former opponents. Bystander affiliations toward aggressors and those toward victims were not likely to occur in the same PC because of this same function of bystander affiliation. We did not measure the anxiety of former opponents directly because the self-directed behavior of dolphins is unknown. Further studies are needed to examine the anxiety reduction function of bystander affiliations for quantitative analyses.

Bystander affiliations might have different functions toward aggressors versus victims, as observed in wolves. In this instance, the function of bystander affiliation toward aggressors may be self-protection by bystanders as further aggression from aggressor against group member is reduced, whereas, the function of bystander affiliation toward victims may protect the victim as renewed aggression from aggressor to victim is reduced^7^. Such differences in the function of bystander affiliation might be affected by the structure of wolf societies^7^. As high-ranked animals receive bystander affiliation more frequently, the authors suggested that bystander affiliation toward aggressors may be shaped by the highly hierarchical structure of wolf society^7^. Contrarily, bystander affiliation toward victim may be affected by the cooperative side of wolf society, because this affiliation offered by bystanders who shared more affiliation relationship with victim^7^. In bottlenose dolphins, bystander affiliations toward both aggressors and victims might have the same function of calming down the tension between opponents. This may be reflected by the social structure of bottlenose dolphins. In previous study using the same data set as this study, the female bottlenose dolphins might have exhibited a relaxed dominance style^24^. Thus, there might have been little necessity for them to pay attention to the action of aggressors, unlike species with despotic dominant styles^2,7^.

Three types of affiliative behaviors (rubbing, contact swimming, and synchronized swimming) were observed in bystander affiliations. These behaviors have also been reported in non post-conflict situation, and these affiliations may function in the formation of social bond^34–36^. In addition, each affiliative behavior may show different functions depending on the situation or sex. For example, contact swimming between females may have function of stress reduction because females are more likely to perform this affiliation in groups that are composed of many males^35^. Specific affiliative behavior types are shown for post-conflict affiliations in chimpanzees, suggesting that they may perform different affiliative behaviors depending on the characteristics of aggression or social relationship^37,38^. Further studies should focus on the occurrence and the functions of each affiliative behavior in post-conflict affiliation to understand the function of affiliation.

## Methods

### Subjects

We observed two captive bottlenose dolphin groups at the Shimonoseki Marine Science Museum and Kagoshima City Aquarium. Shimonoseki Marine Science Museum housed five adult females and one mother–calf male pair during study term. In Kagoshima City Aquarium, we observed five adult females and two calves in total. The observed subjects were sometimes changed (Table S2). More detailed information about the facilities is provided by Yamamoto *et al*.^24,30^. Our study complied with the Ethical Guidelines for the Conduct of Research Animals by Zoo and Aquariums issued by the World Association on Zoos and Aquariums (WAZA) and the Code of Ethics issued by the Japanese Association of Zoos and Aquariums (JAZA). This study was approved by the Shimonoseki Marine Science Museum and Kagoshima City Aquarium.

### Data collection

All dolphins at the Shimonoseki Marine Science Museum were observed between 0830 and 1730 from July 2012 to May 2013 (51 days, approximately 229 h). The dolphins were observed at the Kagoshima City Aquarium between 0900 and 1800 from July 2012 to February 2015 (87 days, approximately 558 h). The observations were adjourned for approximately 30 min during feeding on five or six occasions in both the aquariums. The behavioral data collected were of dolphins that were older than one year.

Definitions of behavior and classification followed that of our previous studies^24,30^. Aggressive behavior included chasing, biting and hitting, and excluded playful interactions. Former opponents were classified as the aggressor who was the last attacker, and the victim was classified as the other former opponent. We recorded whether counter aggression occurred in the aggression and whether the aggressor or victim was a calf. Individuals under the age of six were considered calves, because dolphins tend to remain with their mother until they are three to six years old^21^. We recorded individuals who were closest with the former opponents at the end of aggression. Vocalizations and bubble streams during aggressions were recorded at Shimonoseki Marine Science Museum. We recorded whether dolphins produced whistles or burst-pulses during the aggressions, and whether aggressors and victims emitted bubble streams when whistles or burst-pulses were recorded. Whistles and burst-pulses were identified by the first author listening to the vocalization. If extraneous noises were great, we removed the data of aggression for this recording.

Post-conflict (PC) observations were conducted for 10 min immediately after the original aggression^39^. When renewed aggression between the same former opponents occurred within 1 min after the original aggression, PC observation was cancelled and a new observation was started after the end of aggression. We made only one PC observation of each pair per period between feeding times. If the initiator of post-conflict affiliation between former opponents and bystanders was not clear, the PC was removed for each analysis. During PC, we recorded affiliations initiated by bystanders toward aggressors or victims, affiliation between former opponents and affiliation between group members (not former opponents), and whether the bystander was a calf. In analyzing whether the occurrence of reconciliation affected the occurrence of bystander affiliation, recording of the focal bystander affiliation was extended for the period of reconciliation (for example, if reconciliation continued for 4 min, the period that observation of bystander affiliation was set at 14 min after the aggressions) because former opponents were unable to receive affiliation from bystanders during reconciliation. For the recording of post-conflict affiliation between group members, the target member was a randomly selected individual, who (1) did not initiate bystander affiliation to former opponents, (2) was not the aggressor or victim, and (3) was close to former opponents at the end of aggressions. We recorded whether the targeted member initiated affiliation toward group members during the aggression and PC. We included flipper-rubbing, contact swimming, and synchronized swimming as affiliations^35,40,41^.

Post-conflict attack by aggressors or victims to group members was recorded in the period after bystander affiliation and unaffiliated PC. When bystander affiliation occurred, we recorded the occurrence of PC attack for 10 min after bystander affiliation. We excluded PCs in which there were more than two types of post-conflict affiliation to show the effect of each bystander affiliation. In unaffiliated PCs, the recording period was set as one of three types for each analysis: (1) to compare with MC, the attack was recorded for 10 min after the original aggression; (2) to compare to the period after bystander affiliation toward aggressors, we recorded the attack for 2–12 min after the aggression; (3) to compare to bystander affiliation toward victims, the attack was recorded for 4–14 min after the original aggression. As PC attack was reportedly likely to reduce over time in previous studies on primates^4,5^, we delayed the start time of unaffiliated PC at average times from the end of aggression to the end of bystander affiliation.

Matched-control (MC) observation was set at 10 min from the same time the PC began on the next possible observational day of the corresponding PC. When any aggression occurred within 10 min before scheduled MC, the MC observation was cancelled and restarted after aggressions. If MC observation did not start within 40 min from the time that the corresponding PC started, the MC observation was carried out on the next day. During MC, we recorded whether former opponents attacked group members, and whether the focal member initiated affiliation toward group members.

We recorded the occurrence of synchronized swimming every 10 min to elucidate the affiliation relationship between two individuals. However, the data obtained 10 min after aggressions were excluded from this calculation. The affiliative index was calculated as (*X*_*AB*_/*Y*_*AB*_), where *X*_*AB*_ is the number of periods in which synchronized swimming between individuals *A* and *B* occurred, and *Y*_*AB*_ is the number of periods in which both individuals *A* and *B* were present. The affiliative index in Kagoshima City Aquarium was separately calculated before and after the birth of the second infant because the behavior of the adults differed significantly. The 10-min observational periods totaled 972 (all pairs, respectively) in the Shimonoseki Aquarium, and the average was 823 (min: 243 periods, max: 1173 periods) during the earlier period and 604 (min: 111 periods, max: 1240 periods) during the later period in the Kagoshima City Aquarium. Behavioral and vocal data were collected using both observations and videos (Handycam HDR–CX; Sony, Tokyo, Japan), which were recorded by the first author from underwater windows.

### Statistical analyses

We investigated whether attacks by former opponents to group members increased after the original aggression using the GLMM. The occurrence of attacks toward group members was set as the dependent variable with a binomial error structure and logit link function. The predictor variable was PC or MC. The identities of the aggressor, victim and aquarium were included as random effects. To explore whether bystander affiliation reduced PC attacks on group members, we compared the occurrence of PC attacks in unaffiliated PCs and after bystander affiliations using GLMM with binomial family and logit link functions. The identities of aggressor, victim and aquarium were included as random effects.

Effects of factors on the occurrence of bystander affiliations were investigated using the GLMM with a binomial family (logit link function). The dependent variable was the occurrence of bystander affiliation. The predictor variables were the occurrence of PC affiliation between former opponents before bystander affiliation, the occurrence of bystander affiliation before the other bystander affiliation (i.e., when we investigate bystander affiliation to aggressors, we recorded whether bystander affiliation to the victim occurred before bystander affiliation to the aggressor), counter aggression, the duration of aggression, and whether the aggressor or victim was a calf. The identities of the aggressor and victim, as well as the aquarium, were set as the random effects.

We investigated the effect of affiliative relationships between victims and bystanders on the occurrence of bystander affiliation toward aggressors. The number of bystander affiliations toward aggressors for each victim and potential bystander pair was the dependent variable with a Poisson error structure (log link function) using the GLMM. The affiliative index between the bystander and victim was set as the predictor variable. The opportunity to offer bystander affiliation by each bystander for each aggressor was included as an offset variable. An individual had a chance to offer bystander affiliation to the former opponent when the individual did not fight with the former opponent. For example, in a group composed of individuals A, B, C, and D, individual B had a chance to initiate bystander affiliation toward aggressor A when aggressor A fights with the victim C or D. The number of this chance was used as the opportunity to offer bystander affiliation. As the random effect, we set the identities of the victim, bystander and aquarium. To show the effect of the affiliative relationship between aggressors and bystanders on the occurrence of bystander affiliation toward victims, we ran the GLMM with a Poisson structure (log link function) on the number of bystander affiliations toward victims for each aggressor and potential bystander pair, with the affiliative index between aggressors and bystanders as the predictor variable. The identities of the aggressor and bystander, as well as the aquarium, were set as the random effects.

To explore whether the occurrence of bystander affiliation toward aggressors was affected by affiliative relationship, physical closeness, and whether the bystander was a calf, we ran a GLMM with a Poisson family (log link function) on the number of bystander affiliations toward aggressors for each aggressor and potential bystander pair, with the affiliative index between aggressors and bystanders, the ratio of physical closeness, and whether the bystander was a calf as the predictor variables. The physical closeness ratio was calculated as (the number of times each bystander was closest to former opponents at the end of aggressions)/(the opportunity to offer bystander affiliation). The opportunity to offer bystander affiliation to the aggressor was included as an offset variable. The identities of the aggressor, bystander and aquarium were set as the random effects. Similarly, the number of bystander affiliation toward victims for each victim and bystander pair was set as the dependent variable with a Poisson error structure (log link function) using a GLMM. The affiliative index between victims and bystanders, the ratio of physical closeness with former opponents and whether the bystander was a calf were set as the predictor variables. The opportunity to offer bystander affiliation toward victim was included as the offset variable. The identities of victim, bystander and aquarium were set as the random effects. We ran GLMM in the lme4 package, and compared the full and null models using analysis of variance (ANOVA; car package) to test whether the overall model was improved compared to the null model.

To investigate whether group members was activated to initiate affiliation because they were aroused by aggression, we compared the proportion of attracted and dispersed of PC-MC pair for each former opponent using Wilcoxon matched-pairs signed-ranks exact test (the exactRankTests package). All statistical analyses were conducted in R v. 3.6.0 (R Core Team 2019).

## Supplementary information

Supplementary Information.
