# Supplementary material for: Functions of post-conflict bystander affiliations toward aggressors and victims in bottlenose dolphins
Source: Sci Rep. 2020 Mar 2;10:3776. doi: 10.1038/s41598-020-60423-6 (PMC7052200; doi:10.1038/s41598-020-60423-6)
Supplement: Supplementary file 1 — Supplementary Information. [file 41598_2020_60423_MOESM1_ESM.docx]

Supporting Information

**Functions of post-conflict bystander affiliations toward aggressors and victims in bottlenose dolphins**

Chisato Yamamoto^1,2^, Toshiaki Ishibashi ^3^, Nobuyuki Kashiwagi ^4^, Masao Amano ^2^

^1^ Primate Research Institute, Kyoto University, Inuyama, Aichi 484-8506, Japan

^2^ Graduate School of Fisheries Science and Environmental Studies, Nagasaki University, 1-14 Bunkyo-machi, Nagasaki 852-8521, Japan

^3^ Shimonoseki Marine Science Museum, 6-1 Arcaport, Shimonoseki, Yamaguchi 750-0036, Japan

^4^ Kagoshima City Aquarium, 3-1 Honkoushinmachi, Kagoshima 892-0814, Japan

**Table S1. Sex, year of arrival or birth and mother-calf relationship of dolphins.**

| Shimonoseki Marine Science Museum | | | Kagoshima City Aquarium | | |
| --- | --- | --- | --- | --- | --- |
| Subject | Sex | Mother-Calf | Subject | Sex | Mother-Calf |
| Aruka | Female |  | Naga | Female |  |
| Crown | Male | Calf (Mother: Tiara)  Born: 2009 | Maru | Female |  |
| Kururi | Female |  | May | Female | Calf (Mother: Chiku)  Born: 2013 |
| Patch | Female |  | Milky | Female | Mother (Calf: Lusky) |
| Pearl | Female |  | Tenten | Female |  |
| Rana | Female |  | Chiku | Female | Mother (Calf: May) |
| Tiara | Female | Mother (Calf: Crown) | Lusky | Male | Calf (Mother: Milky)  Born: 2012 |

**Table S2. Observational term and members.** Calf under 1 year was shown an expression in parentheses. In Shimonoseki Marine Science Museum, we observed dolphins in the main and sub pools. Dolphins can move these pools freely. In Kagoshima City Aquarium, we observed dolphins in the main pool. This table was shown members who lived in main pool for each sampling periods.

| Term | Member |
| --- | --- |
| 1. Shimonoseki Marine Science Museum |  |
| July 2012 –May 2015 (51 days) | Aruka, Crown, Kururi, Patch, Pearl, Rana, Tiara |
|  |  |
| 1. Kagoshima City Aquarium |  |
| July – September 2012 (16 days) | Maru, Milky, Chiku, Tenten, Lusky |
| October 2012 – March 2013 (20 days) | Naga, Milky, Chiku, Tenten, Lusky |
| March – April 2013(10 days) | Naga, Milky, Chiku, Tenten, Lusky |
| August 2013 (3 days) | Naga, Milky, Chiku, Tenten, Lusky, May |
| November – December 2013 (7 days) | Milky, Chiku, Tenten, Lusky, May |
| January – February 2014 (7 days) | Naga, Milky, Chiku, Lusky, May |
| March 2014 (3 days) | Milky, Chiku, Tenten, Lusky, May |
| April 2014 (4 days) | Naga, Milky, Chiku, Lusky, May |
| May – July 2014 (12 days) | Naga, May, Milky, Chiku, Lusky |
| August 2014 (2 days) | May, Milky, Tenten, Chiku, Lusky |
| September 2014 (4 days) | Maru, May, Tenten, Chiku |
| October 2014 (3 days) | May, Milky, Tenten, Chiku, Lusky |
| February 2015 (3 days) | Naga, May, Tenten, Chiku, Lusky |

**Table S3. The number of individuals using each analysis.**

|  | Bystander affiliation to aggressors | | Bystander affiliation to victims | |
| --- | --- | --- | --- | --- |
|  | Shimonoseki | Kagoshima | Shimonoseki | Kagoshima |
| Comparing of post-conflict attack between unaffiliated PC and MC | 6 | 6 | 6 | 5 |
| Comparing of post-conflict attack between unaffiliated PC and after bystander affiliation | 6 | 6 | 7 | 6 |
| Effect of factors on the occurrence of bystander affiliation | 7 | 6 | 7 | 6 |
| whether frequency of bystander affiliation was affected by affiliative relationship between bystanders and the enemy | 7 | 7 | 7 | 7 |
| Whether frequency of bystander affiliation was affected by affiliative relationship between former opponent and bystander, physical closeness and whether bystander is calf | 7 | 7 | 7 | 7 |
|  | | | | |
|  | Shimonoseki | | Kagoshima | |
| Occurrence of post-conflict affiliation between group members | 7 | | 7 | |
